# Haplotype structure and heterozygosity around the fragile foal syndrome variant in Swedish Warmblod horses

**DOI:** 10.1111/age.70022

**Published:** 2025-06-16

**Authors:** Michela Ablondi, Susanne Eriksson, Sofia Mikko

**Affiliations:** ^1^ Department of Veterinary Science Università degli Studi di Parma Parma Italy; ^2^ Department of Animal Breeding and Genetics Swedish University of Agricultural Sciences Uppsala Sweden

**Keywords:** balancing selection, extended haplotype, *MFN2*, *PLOD1*

## Abstract

Fragile foal syndrome (FFS) is a disease caused by a recessive lethal missense mutation in the *PLOD1* gene located on ECA2. Despite its harmful effect, a relatively high frequency of FFS carriers was observed in Warmblood breeds spanning from 7.4% in a random sample of Swedish Warmblood breed to 17% in the Hanoverian and Danish Warmblood, indicating potential heterozygous advantage. Balancing selection can be further studied based on haplotype blocks and via detection of heterozygosity‐rich region (ROHet) around the target of selection. In this study we evaluated the presence of haplotype blocks and ROHet on ECA2 in 380 Swedish Warmblood horses. We compared the results of ROHet with the rest of the genome. On average, 11.7 heterozygosity rich regions were identified per horse on ECA2, with no significant difference in numbers and length compared to what was found in other chromosomes. A unique haplotype block containing 28 markers was found in the FFS haplotype, while there were several haplotype blocks in the non‐carrier haplotype. This unique haplotype block mostly spanned the region upstream of the *PLOD1* gene and included the *MFN2* gene. The presence of this extended haplotype, shared by multiple individuals and including both the FFS variant and the *MFN2* gene, suggests that this region may be under selection. While we did not find a clear heterozygosity‐rich region around the FFS variant, the extended haplotype may reflect either a signature of balancing selection or linkage disequilibrium with a positively selected variant in *MFN2*, *PLOD1*, or nearby loci.

Fragile foal syndrome (FFS) is a monogenetic disease caused by a recessive lethal missense point mutation in the *PLOD1* gene located on ECA2 (Monthoux et al., 2015). Most FFS recessive homozygous foals are assumed to be lost by abortion during gestation (Kehlbeck et al., 2024), whereas liveborn affected foals need to be euthanized shortly after birth due to severe skin fragility and joint hyper‐elasticity (Aurich et al., 2019). Despite its harmful effect, a relatively high frequency of FFS carriers was observed in Warmblood breeds spanning from 7.4% in a random sample of Swedish Warmblood breed (SWB) to 17% in the Hanoverian and Danish Warmblood, indicating potential heterozygous advantage (Ablondi et al., 2022; Reiter et al., 2020). This hypothesis of balancing selection was further supported by recent findings in the SWB, where significant positive effects of the FFS allele on movement‐related traits were found (Ablondi et al., 2022). Balancing selection causes various types of signatures in the genome such as: (1) increased diversity around the target of selection; (2) differentiation between populations departing from the same genome‐wide background; and (3) increased linkage disequilibrium around the target of selection (Fijarczyk & Babik, 2015). These signatures frequently appear as distinct haplotype blocks or heterozygosity‐rich regions, where multiple individuals share similar allele combinations, maintained due to selective pressures. Thanks to advances in high‐throughput genotyping technologies, we can now more deeply explore these genomic signatures based on haplotype blocks and heterozygosity‐rich regions detection. Intending to understand if a balancing selection occurs for FFS carriers, this study aims to investigate the presence of haplotype blocks and heterozygosity rich regions (Bizarria dos Santos et al., 2021) around the *PLOD1* gene in SWB horses.

In total, 380 SWB horses born 2010–2011 were genotyped as described in a previous study (Ablondi, Viklund, et al., 2019) using the 670K Affymetrix® Axiom® Equine Genotyping Array. These horses were also genotyped for the XM_001491331: c.2032G>A variant in the *PLOD1* gene by a TaqMan genotyping assay on a StepOnePlus™ instrument (Applied Biosystems, Foster City, CA, USA). The quality control was performed in PLINK v1.90 (Chang et al., 2015), based on the following criteria: minor allele frequency (<0.01), missing genotype per single SNP (>0.10) and missing genotype per individual (>0.10). Heterozygosity‐rich regions (ROHet) were detected using the following settings: minSNP = 15; ROHet = TRUE; maxGap = 10^6^; minLengthBps = 10 kb; maxOppRun = 2; and maxMissRun = 1, as suggested by Bizarria dos Santos et al. (2021), in the R package “detectRUNS” in R v.4.4.1 (Biscarini et al., 2019; R Development Core Team, 2011). ROHet calculation was performed for the whole genome, and ROHet within ECA2 were considered as indicator of heterozygosity rich regions if they were shared by over 25% of the analysed horses. Differences between ECA2 and the rest of the chromosomes in ROHet numbers and length was assessed with ANOVA. The ROHet detection was performed separately in two subgroups of horses, defined as in (Ablondi, Eriksson, et al., 2019), where horses with higher or lower EBV for show jumping than the reference population (mean EBV of 100) were classified as show jumping (SJ) and non‐show jumping (NS) horses, respectively. The average EBV for show jumping trait was significantly higher in SJ horses (average EBV = 125) compared to NS horses (average EBV = 77) based on *t*‐test (*p* < 0.001), corresponding to more than two genetic standard deviations. Based on pedigree, most of the NS horses could be described as horses bred for the dressage discipline. To detect haplotype blocks around the *PLOD1* gene (Gabriel et al., 2002), haplotypes were phased using Beagle 5.4 (Browning et al., 2021), then analysed and visualised using REHH Package (Gautier & Vitalis, 2012) in R. The haplotype carrying the variant allele in the *PLOD1* gene was designated as FFS Haplotype, whereas the haplotype not carrying the variant allele as Normal (*N*) Haplotype. The Ensembl gene annotations EquCab3 was used to identify genomic elements extending 250 kb upstream—and downstream of the potential signatures of balancing selection.

On average, 11.7 ROHet per horse were identified on ECA2, without significant differences between SJ and NS horses. The number and the length of ROHet on ECA2 did not significantly differ from that of ROHet on other chromosomes (data not shown). The detected ROHet on ECA2 had a length ranging between 12.6 kb and 324 489 kb and contained 15–30 SNPs. On average ROHet were 69.5 kb long and included 15.8 SNPs. Specifically, on ECA2, a total of three ROHet were shared by over 25% of the SWB horses within one of the subgroups: one ROHet in the SJ subgroups and two in the NS subgroups (Table 1).

**TABLE 1:** Heterozygosity rich regions on ECA2 shared in over 25% of the Swedish Warmblood breed (SWB) horses within one of the subgroups based on sport discipline (NS and SJ).

Subgroup	Start (bp)	End (bp)	No. SNPs	kb length	Percentage shared (%)
NS	81 932 632	81 972 128	16	39.5	25
NS	101 054 766	101 088 692	12	33.9	32
SJ	42 333 297	42 360 263	3	26.9	27

Abbreviations: NS, non‐show jumping SWB horses; SJ, show jumping SWB horses.

In the case of NS horses, six long non‐coding RNA, five protein coding genes, and one small nuclear RNA were found within the two heterozygosity rich regions shared in more than 25% of the horses. In contrast, in SJ horses, seven protein coding genes, two long non‐coding RNA, and one micro RNA were found. None of the three heterozygosity rich regions on ECA2 shared by more than 25% of the horses overlapped with the *PLOD1* gene.

Compared to the length of runs of homozygosity (ROH) segments found in the SWB breed by (Ablondi, Viklund, et al., 2019), the ROHet detected in this study were much fewer and rarer. This result was expected, since polymorphic sites only account for a small portion of the whole genome (Al Abri et al., 2020). However, the average number of ROHet found in the SWB ECA2 was more than three times higher compared to what found in the Mangalarga Marchador horse breed, which has yet a higher average level of inbreeding based on loss of heterozygosity (0.010) compared to SWB (0.006) (Ablondi, Viklund, et al., 2019; Bizarria dos Santos et al., 2021). The average length of ROHet in the SWB was shorter than in the Mangalarga Marchador horse breed, yet in similar ranges since they found ROHet in the shortest class (0–2 Mb). Nevertheless, we did not find any clear signs of heterozygous‐rich regions close to the FFS variant.

At haplotype level, in the FFS haplotype, a unique haplotype block of 237 kb (exact position 2:39970299–39733606) comprising 27 markers and the *PLOD1* variant (23 upstream and four downstream of the XM_001491331: c.2032G>A variant in the *PLOD1* gene) was found (Figure 1 and File S1). This haplotype block in the FFS haplotype mostly spanned the region upstream of the *PLOD1* gene and included the mitofusin 2 gene (*MFN2*).

**FIGURE 1:**
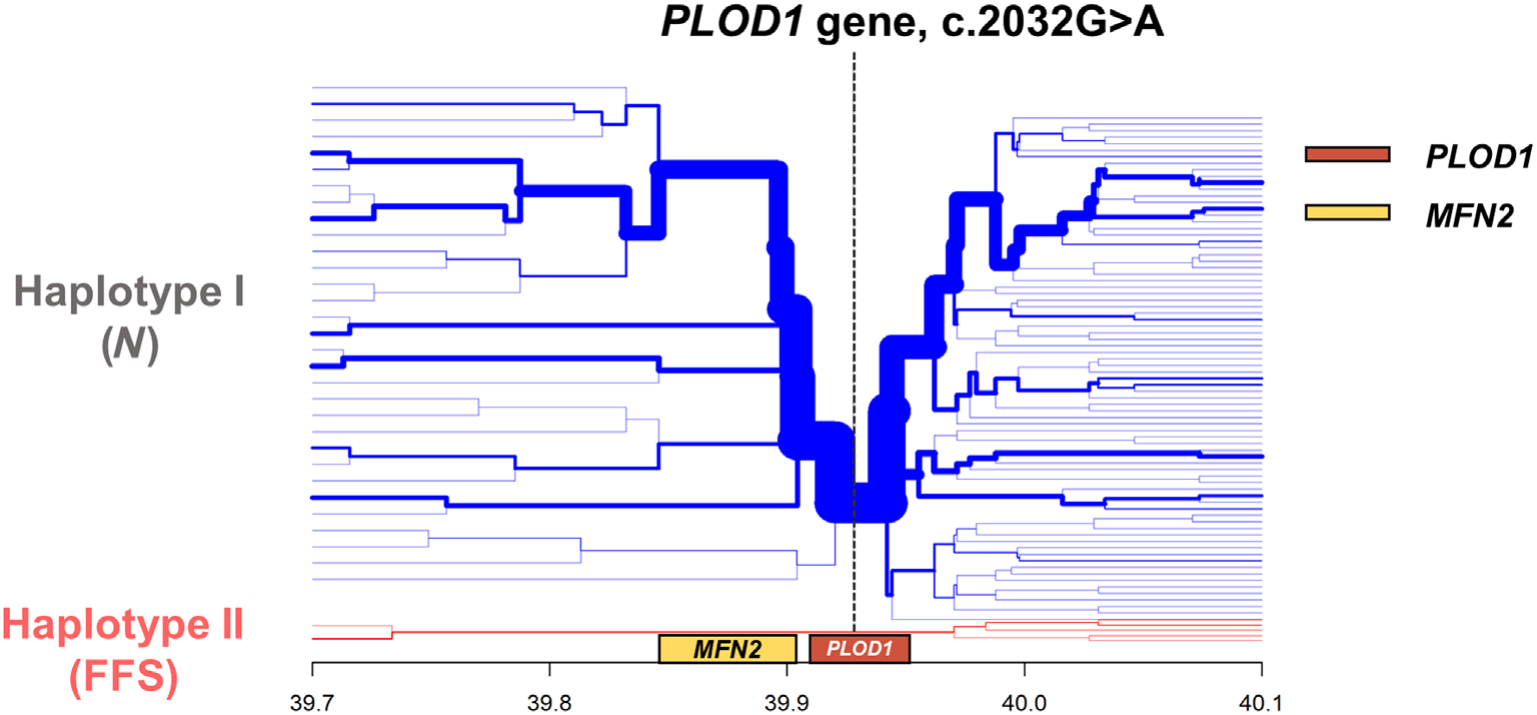
Bifurcation haplotype plot of Haplotype I, which does not carry the fragile foal syndrome (FFS) variant (N), and Haplotype II (FFS), which carries the FFS variant.

In contrast, multiple haplotype blocks were identified in the *N* haplotype within the same genomic region spanned by the FFS haplotype block—specifically, 24 upstream and eight downstream of the FFS locus. This latter result suggested more recombination in the *N* haplotype compared to the FFS haplotype. The *MFN2* is involved in the regulation of vascular smooth muscle cell proliferation and it has been associated with disorders of the peripheral nervous system (Engelfried et al., 2006). Recent studies have reported over 100 mutations in the *MFN2* gene that are associated with Charcot–Marie–Tooth disease type 2A (CMT2A) disease. The CMT2A disease has several symptoms including muscle atrophy and weakness and loss of motor coordination (Abati et al., 2022; Hines et al., 2023). Interestingly, all FFS carrier horses share a haplotype characterised by variant alleles at two SNPs within the *MFN2* gene. At this stage, it remains unclear whether this haplotype is maintained solely due to balancing selection acting on the FFS variant itself, or due to combined effects involving variants in both *MFN2* and *PLOD1* genes. Nevertheless, the presence of this extended haplotype, shared by multiple individuals and including both the FFS variant and the *MFN2* gene, suggests that this region may be important for sport horses. While we did not find a clear heterozygosity‐rich region around the FFS variant, the extended haplotype may reflect either a signature of balancing selection or linkage disequilibrium with positively selected variants within the FFS haplotype block. In addition, we can exclude the possibility that the haplotype block in the FFS haplotype is the result of an overrepresentation of a popular parent since the carrier horses were born from 13 different stallions and 28 mares. Additional studies investigating the presence of the FFS haplotype also in other breeds may be useful to further understand the potential role of this haplotype in sport horses. This study provides the first characterisation of an FFS haplotype block surrounding the *PLOD1* gene in the SWB breed which may be another sign of presence of balancing selection for this region or linkage disequilibrium with a positively selected variant within the FFS haplotype block.

## FUNDING INFORMATION

Open access funding provided by Swedish University of Agricultural Sciences. This research was funded by Formas, Grant Number 2020‐01333.

## AUTHOR CONTRIBUTIONS

**Michela Ablondi:** Writing – original draft; formal analysis; visualization; conceptualization; methodology. **Susanne Eriksson:** Supervision; writing – review and editing; conceptualization; data curation. **Sofia Mikko:** Project administration; supervision; writing – review and editing; funding acquisition; conceptualization.

## CONFLICT OF INTEREST STATEMENT

The authors declare no conflict of interest.

## Supporting information


File S1.

## Data Availability

Restrictions apply to the availability of these data, which were used under licence for this study. Data are available from the authors with the permission of the Swedish Warmblood Association.

## References

[age70022-bib-0001] Abati, E. , Manini, A. , Velardo, D. , Del Bo, R. , Napoli, L. , Rizzo, F. et al. (2022) Clinical and genetic features of a cohort of patients with MFN2‐related neuropathy. Scientific Reports, 12(1), 6181. 35418194 10.1038/s41598-022-10220-0 PMC9008012

[age70022-bib-0002] Ablondi, M. , Eriksson, S. , Tetu, S. , Sabbioni, A. , Viklund, Å. & Mikko, S. (2019) Genomic divergence in Swedish Warmblood horses selected for equestrian disciplines. Genes, 10(12), 976. 31783652 10.3390/genes10120976 PMC6947233

[age70022-bib-0003] Ablondi, M. , Johnsson, M. , Eriksson, S. , Sabbioni, A. , Gelinder Viklund, Å. & Mikko, S. (2022) Performance of Swedish Warmblood fragile foal syndrome carriers and breeding prospects. Genetics Selection Evolution: GSE, 54, 4. 35062868 10.1186/s12711-021-00693-4 PMC8783495

[age70022-bib-0004] Ablondi, M. , Viklund, Å. , Lindgren, G. , Eriksson, S. & Mikko, S. (2019) Signatures of selection in the genome of Swedish Warmblood horses selected for sport performance. BMC Genomics, 20(1), 717. Available from: 10.1186/s12864-019-6079-1 31533613 PMC6751828

[age70022-bib-0005] Al Abri, M.A. , Holl, H.M. , Kalla, S.E. , Sutter, N.B. & Brooks, S.A. (2020) Whole genome detection of sequence and structural polymorphism in six diverse horses. PLoS One, 15(4), e0230899. Available from: 10.1371/journal.pone.0230899 32271776 PMC7144971

[age70022-bib-0006] Aurich, C. , Müller‐Herbst, S. , Reineking, W. , Müller, E. , Wohlsein, P. , Gunreben, B. et al. (2019) Characterization of abortion, stillbirth and non‐viable foals homozygous for the warmblood fragile foal syndrome. Animal Reproduction Science, 211, 106202. 31785623 10.1016/j.anireprosci.2019.106202

[age70022-bib-0007] Biscarini, F. , Cozzi, P. , Gaspa, G. & Marras, G. (2019) *detectRUNS: an R package to detect runs of homozygosity and heterozygosity in diploid genomes* [Internet]. Available from: https://cran.r‐project.org/web/packages/detectRUNS/vignettes/detectRUNS.vignette.html#references. Accessed September 15, 2024.

[age70022-bib-0008] Bizarria dos Santos, W. , Pimenta Schettini, G. , Fonseca, M.G. , Pereira, G.L. , Loyola Chardulo, L.A. , Rodrigues Machado Neto, O. et al. (2021) Fine‐scale estimation of inbreeding rates, runs of homozygosity and genome‐wide heterozygosity levels in the Mangalarga Marchador horse breed. Journal of Animal Breeding and Genetics, 138(2), 161–173. Available from: 10.1111/jbg.12508 32949478

[age70022-bib-0009] Browning, B.L. , Tian, X. , Zhou, Y. & Browning, S.R. (2021) Fast two‐stage phasing of large‐scale sequence data. American Journal of Human Genetics, 108(10), 1880–1890. 34478634 10.1016/j.ajhg.2021.08.005 PMC8551421

[age70022-bib-0010] Chang, C.C. , Chow, C.C. , Tellier, L.C. , Vattikuti, S. , Purcell, S.M. & Lee, J.J. (2015) Second‐generation PLINK: rising to the challenge of larger and richer datasets. GigaScience, 4(1):7. Available from: 10.1186/s13742-015-0047-8/2707533 25722852 PMC4342193

[age70022-bib-0011] Engelfried, K. , Vorgerd, M. , Hagedorn, M. , Haas, G. , Gilles, J. , Epplen, J.T. et al. (2006) Charcot‐Marie‐Tooth neuropathy type 2A: novel mutations in the mitofusin 2 gene (MFN2). BMC Medical Genetics, 7(1), 53. Available from: 10.1186/1471-2350-7-53 16762064 PMC1524942

[age70022-bib-0012] Fijarczyk, A. & Babik, W. (2015) Detecting balancing selection in genomes: limits and prospects. Molecular Ecology, 24(14), 3529–3545. Available from: 10.1111/mec.13226 25943689

[age70022-bib-0013] Gabriel, S.B. , Schaffner, S.F. , Nguyen, H. , Moore, J.M. , Roy, J. , Blumenstiel, B. et al. (2002) The structure of haplotype blocks in the human genome. Science, 296(5576), 2225–2229. Available from: 10.1126/science.1069424 12029063

[age70022-bib-0014] Gautier, M. & Vitalis, R. (2012) Rehh: an R package to detect footprints of selection in genome‐wide SNP data from haplotype structure. Bioinformatics, 28(8), 1176–1177. Available from: 10.1093/bioinformatics/bts115 22402612

[age70022-bib-0015] Hines, T.J. , Bailey, J. , Liu, H. , Guntur, A.R. , Seburn, K.L. , Pratt, S.L. et al. (2023) A novel ENU‐induced Mfn2 mutation causes motor deficits in mice without causing peripheral neuropathy. Biology (Basel), 12(7), 953. 37508383 10.3390/biology12070953 PMC10376023

[age70022-bib-0016] Kehlbeck, A. , Blanco, M. , Venner, M. , Freise, F. , Gunreben, B. & Sieme, H. (2024) Warmblood fragile foal syndrome: pregnancy loss in warmblood mares. Equine Veterinary Journal, 57(4), 915‐923. Available from: 10.1111/evj.14435 39539185

[age70022-bib-0017] Monthoux, C. , de Brot, S. , Jackson, M. , Bleul, U. & Walter, J. (2015) Skin malformations in a neonatal foal tested homozygous positive for warmblood fragile foal syndrome. BMC Veterinary Research, 11(1), 12. 25637337 10.1186/s12917-015-0318-8 PMC4327794

[age70022-bib-0018] R Development Core Team . (2011) R: a language and environment for statistical computing. Vienna: R Foundation for Statistical Computing.

[age70022-bib-0019] Reiter, S. , Wallner, B. , Brem, G. , Haring, E. , Hoelzle, L. , Stefaniuk‐Szmukier, M. et al. (2020) Distribution of the warmblood fragile foal syndrome type 1 mutation (PLOD1 c.2032G>a) in different horse breeds from Europe and the United States. Genes, 11(12), 1518. 33353040 10.3390/genes11121518 PMC7766603

